# Delaying rewards has greater effect on altruism when the beneficiary is socially distant

**DOI:** 10.1371/journal.pone.0170387

**Published:** 2017-02-14

**Authors:** Jerzy Osiński, Adam Karbowski

**Affiliations:** 1 University of Warsaw, Faculty of Psychology, Warsaw, Poland; 2 Warsaw School of Economics, Warsaw, Poland; University of Kansas Medical Center, UNITED STATES

## Abstract

Based on the assumption that social distance and time are dimensions of psychological distance important for altruistic choices it was predicted that enhancement of altruism due to delaying rewards when choosing between a reward for oneself and for another person would be more pronounced the greater the social distance between the subject and another person. In order to test this hypothesis, social discounting using hypothetical monetary rewards and manipulation of social distance and reward delay was measured in a group of 161 college students. The results indicate that delaying rewards increasingly enhances preference for altruistic choices as the social distance between subject and beneficiary grows.

## Introduction

According to Rachlin and Jones [1], both temporal and social discounting are associated with the “extension of the self”: temporal discounting depends on how extended the self is in time, while social discounting on the extension of the self in the social sphere. Self-control and altruism require a self that is extended in both the temporal and social dimension.

The fact that the subjective value of a reward is a hyperbolic function of delay (for a review, see Green and Myerson [2]) can be explained in terms of preference for the “present self” over the “future self”, which in turn may suggest that people tend to treat their future self as a different person [3]. Consistently with this point of view, Yi et al. [4] demonstrated that by manipulating the dimension of time we can influence the rate of social discounting: postponing a reward for oneself and for another person by an equal amount of time shifts preferences towards the reward for another person. In the light of the ideas of Rachlin and Jones [1], this effect can be readily explained by discounting of delayed rewards: the same delay should result in greater decrease in the subjective value of a reward in the case of a reward for oneself than for another person. This interpretation has been supported further by the findings of Pronin et al. [3], who reported that choices between an immediate and a delayed reward for oneself were more likely to be impulsive than choices made between immediate and delayed reward for another person and choices between two delayed rewards for oneself. Furthermore, results indicate that the reward for oneself is discounted over time faster than the rewards for “us”, regardless of whether choices are “we now—we later” or “me now—we later” [5, 6].

Drawing on the extended self concept [1], we can expect that delaying rewards when the choice is between a reward for oneself and one for another person enhances altruism and the extent of that enhancement is dependent on social distance between the subject and the other person (beneficiary). If recipients ranked high on the social distance list (cf. Yi et al.[4]) are part of the extended self to a greater extent than those occupying lower positions, the same reward delay should equally devalue the reward for oneself and a close person, and produce greater discounts in the value of reward for oneself than for a distant person. These considerations are also consistent with Construal Level Theory [7, 8], according to which the social and temporal distance are examples of psychological distance expressed as a degree of abstraction/reality of events—an increase in the distance (and therefore a decrease in the reality) in one dimension results in the decline of the second dimension.

Charlton et al. [6] present a formal analysis of the problem of connecting the effect of social distance and the delay, in which part of a hyperbolic function of the discount includes two variants of their combinations—multiplicative and additive. Both models predict that when the social distance is 0 (as it practically is in the case of a closest person) the discounting rate is determined solely by the delay. As a result, the reward for the closest person is discounted at the same rate as the reward for oneself, and that means that the situation of the choices between the reward for oneself and the reward for the closest person actually becomes a choice between two rewards for oneself delayed for the same stretch of time. However, only the additive model shows that when the social distance is greater than 0, the slope of the discount curve will be lower than in the case of the curve for oneself.

The purpose of the present study was to test the prediction that when faced with a choice between a delayed reward for oneself and an equally delayed reward for another person, the subjective value of a reward for a close person will not be affected by delay, while subjective value of a reward for a distant person should increase as a function of delay.

## Materials and methods

### Participants

Participants were 161 students of two Polish universities (University of Warsaw and Warsaw School of Economics) aged 19 to 30 years (M = 21.94, SD = 2.5), 84 of them female.

The participants did not receive any form of remuneration.

### Materials

A software application was used to determine the subjective value of a reward for another person. In general terms, the study design was similar to that applied by Yi et al. [4], but since our goal was not a replication of this study, we have introduced some changes. Above all, considering the qualitative rather than quantitative effect of delay obtained in the studies by Yi et al. [4], we used a steeper time scale (see Discussion), and moreover, we took into account two sizes of reward.

As in the study by Yi et al. [4], participants were asked to imagine a list of 100 people ranked in terms of social distance from the participant (person no. 1 was to be the closest and person no. 100 only a passing acquaintance). Then, participants made hypothetical choices between a smaller monetary reward for themselves (option A) available after a specific delay (immediately, tomorrow, in a month, in five years), and a greater reward for a person ranked 1, 5, 20, 60 and 100 on the imagined list (option B), delayed by the same amount of time as the reward for themselves (Table 1 presents the combinations of delay and social distance). The amount of money in option B was fixed, while in option A it changed according to a titration algorithm described by Holt et al. [9], i.e. in the first choice it was half of the amount in option B, and in subsequent steps it was increased or decreased depending on a given participant’s choices. If in the first choice the participant preferred option A (amount for himself), in the second choice the amount of option A was reduced by half; if he chose option B (amount to be shared), the amount of option A was increased. With each choice the amount in option A was titrated by half of the preceding change. The amount A adjusted following the sixth choice was treated as the equivalent of the reward in option B (the so-called indifference point), i.e. its subjective value. Each participant made a total of 120 choices: 5 (beneficiary ranks in option B on the social distance axis) x 4 (delay) x 6 (titration). The sequence in which tasks were presented was balanced in terms of social distance and fixed in terms of delay value (increasing sequence). The amount in option B was PLN 900 (n = 86) or 70,000 (n = 75) (at the time of the study 1 PLN = ca. $0.3).

**Table 1:** Mean subjective value of a reward for another person (expressed as the proportion of its objective value) depending on delay time and social distance.

	REWARD DELAY							
SOCIAL DISTANCE	*Immediately*		*1 day*		*1 month*		*5 years*	
	M	SD	M	SD	M	SD	M	SD
*1*	.740	.303	.764	.290	.753	.299	.742	.288
*5*	.498	.334	.523	.333	.532	.330	.529	.325
*20*	.253	.276	.281	.302	.307	.310	.319	.313
*60*	.143	.219	.151	.230	.165	.248	.208	.271
*100*	.100	.208	.111	.215	.124	.230	.162	.262

### Procedure

The study was approved by the Ethics Committee of the Faculty of Psychology at the University of Warsaw. Due to the non-jeopardizing character of the study, the committee did not require written consent to be obtained from the participants. The consent form appeared on the first screen of the application used for the study, the participants familiarized themselves with the form and passed to the next screen only if they wanted to participate in the study. Giving the answer on the next screen documented consent to participate in the study.

Participants completed the study individually, in the presence of experimenter. No time limit was imposed (in practice 15 minutes was the most participants took). Participants were not paid for participation.

## Results

All observations were valid and have been included in statistical analysis. Table 1 presents mean subjective value of a reward for another person (expressed as the proportion of its objective value) depending on time delay and social distance. Like Yi et al. [4], for statistical verification of the effect of the postponement in terms of the rate of social discounting we used the measure proposed by Myerson et al. [10]–area under the discounting curve (AUC) obtained by drawing lines between successive points representing subjective value of reward for individuals occupying successive ranks on the social distance axis. High values of the area indicate shallow discounting, and thus low egoism. MANOVA 4 (delay) x 2 (amount of reward) revealed the main effect of delay (F(3, 157) = 8.599; p < 0,001; η_p_^2^ = .147; M ± SEM: immediately .217 ± .016. tomorrow .233 ± .017. in a month .248 ± .018, in five years .276 ± .019). Helmert contrast tests yielded significant differences within each pair of measurements. There was no effect of amount of reward (F(1, 159) = 1.025; p = .313; M ± SEM: 900 PLN—.260 ± .023, 70000 PLN—.226 ± .025), or the effect of interaction between delay and amount of reward (F(3, 157) = .248; p = .862).

**Fig 1:**
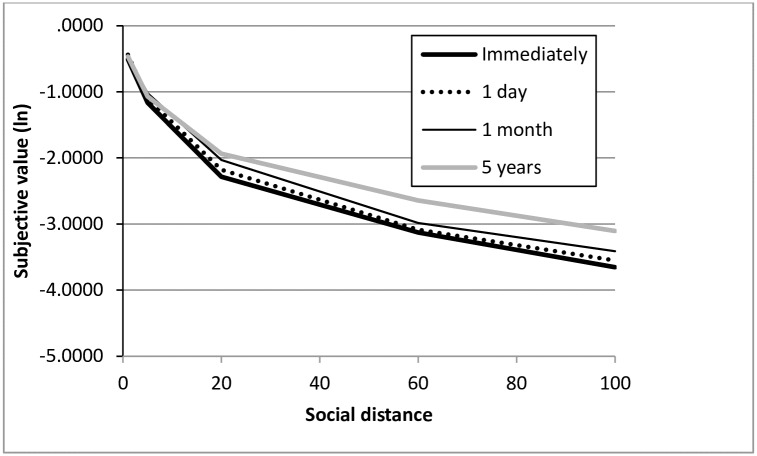
Mean log-transformed subjective value of a delayed reward as a function of social distance. The area under a curve consisting of empirical data corresponds to the area-under-the curve measure widely utilized in the discounting literature.

**Fig 2:**
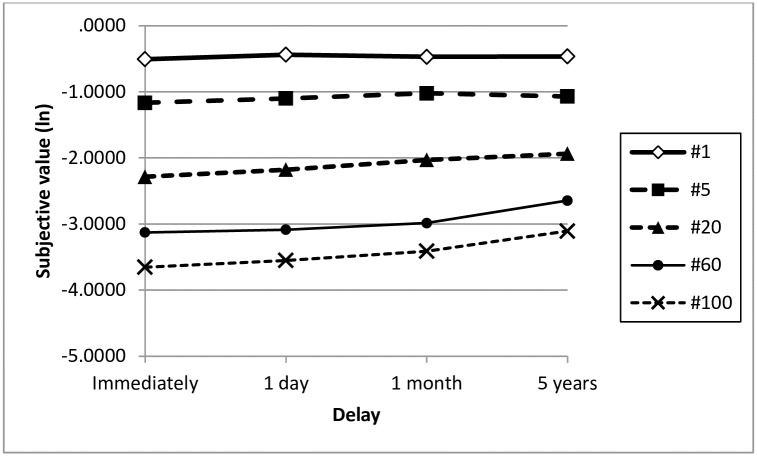
Mean log-transformed subjective value of a reward for another person as a function of delay.

However, as expected, the increase in the appeal of a reward for another person as a function of delay was steeper the greater the social distance between the beneficiary and subject (See Table 1, Figs 1 and 2). MANOVA 5 (social distance) x 4 (delay) x 2 (amount of reward) executed on log values of indifference points expressed as the proportion of a reward for another person revealed main effects of social distance (F(4, 156) = 172.87; p < .001, η^2^ = .816) and delay (F(3, 157) = 13.027; p < .001, η_p_^2^ = .199), and confirmed the expected interaction between social distance and delay (F(12, 148) = 2.476; p = .006, η_p_^2^ = .167). The analysis of simple effects revealed a lack of effect of delay on the subjective value of a reward for person ranked 1 on the social distance dimension (F(3, 158) = .894; p = .446, η_p_^2^ = .017), a marginally significant effect for person ranked 5 (F(3, 158) = 2.624; p = .052, η_p_^2^ = .047), as well as statistically significant effects for persons ranked 20, 60 and 100 (no. 20: F(3, 158) = 4.621; p = .004, η_p_^2^ = .081; no. 60: F(3, 158) = 6.822; p < .001, η_p_^2^ = .115; no. 100: F(3, 158) = 10.458; p < .001, η_p_^2^ = .166). Helmert contrast tests (comparing the mean from each measurement with the mean from the subsequent measurement) performed for positions 20, 60 and 100 showed that the subjective value of a reward rises with each delay increment. No other interactions were detected, however, a statistically significant (though weak) main effect of the amount of a reward was present (F(1, 157) = 9.93; p = .002, η_p_^2^ = .06; M ± SEM for indifference points before logarithmic transformation: PLN 900: .391 ± .022, PLN 70,000: .348 ± .024). There was no effect of sex (F(1, 157) = .88; p = .35).

## Discussion

Our results have confirmed the findings reported by Yi et al. [4] that delaying a reward for oneself and for another person by the same amount of time results in shallower social discounting. Validation was obtained on a group of participants from a different population (Polish students vs. American students in the study by Yi et al.), using different delay periods (day, month, 5 years vs. 6 months, 12 months and 3 years, 6 years) and different reward sizes (PLN 900 and PLN 70,000, which at the time of the study was equivalent to approx. $300 and $23,000 vs. $100 in the Yi et al. study). It is worth noting that the effect was present regardless of the amount of reward.

However, the main purpose of the present study was to test the prediction derived from the concept of extended self [1] that the increase in altruism when delaying rewards in the choice between a reward for oneself and for another person is more pronounced the greater the social distance between the subject and the another person. As demonstrated in Table 1 showing mean subjective values of rewards for another person, the results are a precise match for the prediction: when the recipient is the closest person, the level of altruism is high regardless of delay, while when the beneficiary is a distant person, the level of altruism is lower, but increases with the delay. This leads to the conclusion that, as shown by Yi et al. [4], the decrease in the rate of social discounting attributable to the delay of the reward corresponds mainly to the slower discounting of delayed rewards for distant people.

Our results become part of the discussion based on behavioural economics on the way the influence of social distance and delay are linked. Results corresponding to the additive model [6], although only in the range of one dimension of discounting, were obtained by Green et al. [11], who found that the rate of discounting in time decreases when both rewards, smaller sooner reward and larger later reward, have the same value of delay added (adding the same delay favours reversing the preferences from the reward available sooner to the reward available later). Similar results have also been produced in the study by Osiński et al. [12] focused on social discounting: shifting two rewards, the smaller “socially closer” one (namely a reward for a person who is closer to you in terms of social distance) and the larger one, “more distant” in the social sense, by the same social distance from the subject invariably led to a stronger preference for the “socially more distant” reward (and so the rate of social discounting decreased). An analogous relationship, in accordance with the additive model, can be expected by summing the social distance with the delay—adding the delay to social distance (or vice versa—increasing the social distance towards the delayed reward) should reduce the rate of discounting. Evidence confirming these expectations can be found in the work by Kim et al. [13] who demonstrated that preference for a more delayed reward increased when decisions were made on behalf of a socially distant as opposed to a close person. Also, Charlton et al. [6] in comparing the rate of discounting in the situation of choices, “me now—me later” vs. “we now—we later” (“we” meant the subject and nine unfamiliar people)–found that adding a social component to the choices between the immediate and delayed reward moved preferences towards the delayed reward, although you can argue about whether the addition of other beneficiaries to the self actually increases the social distance towards the reward. In our study, the opposite—we added the same amount of delay to the reward for oneself and reward for another person, and the effect of this treatment was a reduction in the rate of social discounting, predicted by the additive model. Our results show that the decrease in the rate of discounting in one dimension (here social) occurs relatively proportionally to distance growth in the other dimension.

In addition, we obtained other results that are worth discussing in relation to the literature on discounting. Firstly, in contrast to Yi et al. [4], who showed the qualitative effect of the delay (i.e. social discounting was slower in the case of delayed rewards rather than immediate, but the more or less delayed rewards were discounted at the same rate), in the present study we found a quantitative effect (rate of social discounting gradually decreased with increasing delay). The difference in the results of these two studies seems to confirm the interpretation presented by Yi et al. [4] that the lack of expected quantifying effect of the delay, based on the fact, well-documented in temporal discounting studies, that people experience time non-linearly (adding the same unit of time to a small delay has a greater impact on the subjective value of the delay than adding to a large delay) so that for the quantitative effect of increments to occur delay must be proportionally increasing. At the same time, in Yi et al.'s [4] study delay representing the same quality category size (months or years) and a small, two-time, increase in time were compared, while in our study the delays represented other categories of units of time (day, month, year), and therefore also a significantly higher growth in delay.

Secondly, we have confirmed, as reported in the literature [14, 15] a reversed amount effect for social discounting (rate of discounting is lower for small rewards), which is analogous to the results obtained for probabilistic discounting (e.g. Green et al. [16]). “Reversed” refers to the widely discussed “amount effect” characteristic for the temporal discounting and involving a higher rate of discounting for smaller rewards than for larger ones (for a comprehensive review, see [2]). It is not clear what links social discounting with probabilistic, and therefore differentiates them from temporal discounting. As suggested by Rachlin and Jones [15] it may be a case of potential benefits to the decision maker (because of common interests with the recipient) which are probabilistic in nature, where the probability of their reference is greater, the smaller the social distance.

In conclusion, the most important of the results we have achieved shows that future altruism depends more on the length of delay, the greater the social distance between subject and recipient. We suggest that this effect was due to differences in the rate of discounting of delayed rewards for themselves and for others—rewards for others are discounted in time slower the greater the social distance separating the person from the subject. This explanation, however, requires the support of more test results.

## Supporting information

S1 File Data description. (TXT)

S2 File Data. (TXT)

## References

[1] Rachlin H, Jones BA. The extended self In: Madden GJ, Bickel WK, editors. Impulsivity: theory, science, and neuroscience of discounting. Washington: American Psychological Association; 2009 pp. 411–439.

[2] Green L, Myerson J. A discounting framework for choice with delayed and probabilistic rewards. Psychol. Bull., 2004;130:769–772. 10.1037/0033-2909.130.5.769 15367080 PMC1382186

[3] Pronin E, Olivola C, Kennedy K. Doing unto future selves as you would do unto others: Psychological distance and decision making. Pers. Soc. Psychol. B., 2008;34:224–236. 10.1177/0146167207310023 18156588

[4] Yi R, Charlton S, Porter C, Carter AE, Bickel WK. Future altruism: social discounting of delayed rewards. Behav. Process., 2011;86:160–163. 10.1016/j.beproc.2010.09.003 PMC3552492 20950676

[5] Bickel WK, Jarmolowicz DP, Mueller ET, Franck CT, Carrin C, Gatchalian KM. Altruism in time: social temporal discounting differentiates smokers from problem drinkers. Psychopharmacology 2012;224:109–120. 10.1007/s00213-012-2745-6 22644127 PMC10449014

[6] Charlton SR, Yi R, Porter C, Carter AE, Bickel W, Rachlin H. Now for Me, Later for Us? Effects of Group Context on Temporal Discounting. Behav. Dec. Making 2013;26:118–127. 10.1002/bdm.766 PMC3639500 23641123

[7] Maglio S J, Trope Y, Liberman N. Distance From a Distance: Psychological Distance Reduces Sensitivity to Any Further Psychological Distance. J. Exp. Psychol. Gen., 2012;142:644–657. 10.1037/a0030258 23025560

[8] Trope Y, Liberman N. Temporal construal. Psychol. Rev., 2003;110:403–420. 12885109 10.1037/0033-295x.110.3.403

[9] Holt DD, Green L, Myerson J. Is discounting impulsive? Evidence from temporal and probability discounting in gambling and non-gambling college students. Behav. Process., 2003;64:355–367. 10.1016/s0376-6357(03)00141-4 14580704

[10] Myerson J, Green L, Warusawitharana M. Area under the curve as a measure of discounting. J. Exp. Anal. Behav., 2001;76:235–243. 10.1901/jeab.2001.76-235 11599641 PMC1284836

[11] Green L, Myerson J, Macaux EW. Temporal discounting when the choice is between two delayed rewards. J. Exp. Psychol.-Learn. Mem. Cogn., 2005;31:1121–1133. 10.1037/0278-7393.31.5.1121 16248754

[12] Osiński J, Karbowski A, Ostaszewski P. Social discounting: Choice between rewards for other people. Behav. Process., 2015;115:61–63. 10.1016/j.beproc.2015.02.010 25747110

[13] Kim H, Schnall S, White MP. Similar Psychological Distance Reduces Temporal Discounting. Pers. Soc. Psychol. B., 2013; 39:1005–1016. 10.1177/0146167213488214 23653066

[14] Ostaszewski P, Osiński J. Social discounting of monetary rewards. Eur. Psychol., 2011;16:220–226.

[15] Rachlin H, Jones BA. Social discounting and delay discounting. J. Behav. Decis. Making 2008;21:29–43.

[16] Green L, Myerson J, Ostaszewski P. Amount of reward has opposite effects on the discounting of delayed and probabilistic outcomes. J. Exp. Psychol. Learn., 1999;25:418–427. 10.1037//0278-7393.25.2.418 10093208

